# Extracts from Our Correspondents

**Published:** 1871-11

**Authors:** 


### EXTRACTS FROM OUR CORRESPONDENTS.

Some time since we requested our Associate and Corresponding Editors, as well as other friends, to furnish us "items" upon every subject having for their object the dignity and wellfare of the Profession. In response to this, we lay before the readers of THE COMPANION a number of extracts and paragraphs, exhibiting the sentiments and views of some of our editors and friends. Our object is to furnish a journal to promote the best interests of the Profession. We wish to benefit every section, and to show that what is injurious in one locality, is equally so in any other. We earnestly appeal to our friends to aid us in the effort we are making to bring the Profession to the true principles of the ethics. It is not our desire or wish to monopolize our editorial space; and hence, we have opened it to our friends, everywhere, that they may, through it, reach the Profession. In this way we feel assured every topic of interest to the Profession, will be duly considered and discussed:

"Your editorial on morals and ethics is well calculated and must do an immense amount of good. I hope you will continue to urge upon the profession the necessity of a high standard of morals and ethics. It is absolutely necessary for the honor of the profession, the advancement of science, and the safety of the sick."

"When it becomes dishonorable to vindicate the truth or of doubtful policy, I desire to abandon the profession."

"Stick to correct principle and you will succeed. Forsake principle for compromises and policies, and your enterprise will go under like all countries and organizations, political or religious, that laid aside principle for a policy."

"Stand firm to principle. The truth never looses by time or investigation. Men of character can't afford to advocate falsehood long, and those who have no character can't throttle the truth or do honest men much injury."

"I WAS gratified in reading, in the October Number of "Our" COMPANION, to see your editorial in response to "A Quarrel Ended." That editorial, taken in connection with the pamphlet issued by the Fulton County Medical Society, ought to put to blush the parties who, with so high an hand, manipulated and overrode the action of the Georgia Medical Association, at Americus. After reading these, I was amazed at the reckless folly of the so-called rescinding resolution. In that resolution it is asserted and charged that all the action against the Atlanta Medical College on the part of the Association was in consequence of, and due to, *personal feeling*. In your editorial you show, in the most amiable spirit, that the difficulty existed between the faculty and the trustees of the college long before the question ever reached the Association. You show also the true nature and character of the amendment, and what were the practical workings of this "fraudulently procured" amendment, according to the Trustees, as manipulated by the Faculty. That the amendment was a violation of ethics and its authors deserving of professional censure, cannot be doubted; and the Georgia Medical Assciation, composed of honest men, was bound to notice and condemn it. For this they were grossly insulted, and the Association itself declared not to have been the Association but a body of envious and dishonorable physicians seeking the ruin of a medical instiution! The Association was right in demanding an apology. The affront put upon the Association could not be wiped out in any other way, and the—what shall I term it?—*singular* effort made by the Faculty to *deceive* the Association by tendering, on two occasions, apologies which they did not themselves regard as apologies, only added wrong to wrong and insult to insult.

"As I understand it, the Association made three demands upon the old Faculty at Savannah, *after* the repeal of the Amendment, which demands arose only in consequence of the unprofessional and insulting language used in the memorial, which the gentlemen of the Association were bound to do or forfeit their self-respect and honor.

"'1st. That the new Faculty repudiate the acts of the old faculty under the Amendment.

"'2d. That "a distinct and unequivocal" apology be made the Association by the faculty in reference to the language used in the memorial.

"'3d. That this apology should be circulated as widely as had been the memorial, viz, in the newspapers of the State.'

None of these requirements were met and satisfied, but instead a "packed Association" declared not only that they were unnecessary and unjust, but that they were based, not upon principle and professional honor, but were the offspring of "*personal feeling*" and therefore should be forever obliterated from the archives of that body! In other words, that action would seem to assert that there had never been any amendment at all—that there had never been any rupture and hostility between the faculty and trustees, nor no ethical reason for the condemnation of the faculty by the Association!! But enough! How shall honorable gentlemen characterize such proceedings? For myself I say—well, bosh!"

"We hope and trust every editor (and, indeed, every friend) of The Companion will enter heartily into the spirit of extending the circulation of 'our medical adviser' and best friend. Dr. Lewis deserves the gratitute of every medical gentleman who desires to see a good medical journal built up in our Section. I endorse what he says, and earnestly and affectionately urge and entreat the friends of truth, honor, principle and science, to make a long pull, a strong pull, and a pull altogether, in the worthy and noble enterprise, in which you and your gentlemanly publisher are so energetically at work."

We can but "endorse" these kind words. Will not our friends "endorse" them, and at once show their "faith by their works?" Whatever is done for THE COMPANION is done for the Profession. Very little time—very little trouble, on the part of its genuine friends, will be required to make up a club in every part of the country. Will our friends—our true friends—not aid in so good a cause at the expense of so small an outlay of time and trouble?

"WE all know how the profession of medicine was honored and respected a few years ago. This respect is still shown the profession of other countries—unfortunately in ours, the profession has been thrown "in the ditch," and is regarded as a shrewd system of fraud and humbuggery. In fact, the whole world—ignorant and learned—are arrayed against it, sneer and deride it—and refuse to extend to it the honor, or courtesy, of regarding it as one founded upon science. Why is all this? One reason is, the profession has insulted the intelligence of the country by ignoing the claims of the people to understand the laws controlling the human body in health and disease, instead of instructing them. Make a people understand the truths of medicine, and quackery will cease from the land."*

While we admit that the people have not been properly educated, as to the objects and aims of the profession, and in consequence thereof, have become subject to all the ills of charlantry, yet there is, in medicine as well as religion, too much disposition to cast these "pearls before swine." "A little learning is a dangerous thing," a fact too often illustrated by the ignorant, unlettered clout, throwing aside the "shovel and pick" to play the role of Doctor. If the people were *thoroughly* educated, as to the objects, aims and laws which should control medicine and, therefore, capable of judging correctly of medical men and science, then, indeed, quackery would cease, and science be enthroned in the hearts of all. These can alone be accomplished by having the truth brought prominently before the people in the secular press. When the value of law. as embodied in medical ethics, is duly understood and appreciated, then medicine will take its former hold upon the affections of the people. Why the secular press should, politically, encourage organization, and blatantly assert that "right and truth" should prevail *socially*, and then quietly ignore and positively sneer at medical men because they desire the same wise organization in science, is marvelous. Are governments and society of more importance than the human.body itself? Is life of so little value that everybody shall attempt to destroy the men with the means in their hands of preserving it? If charlantry is a crime in politics and religion, why not, when it has for its object the destruction of human life? If organization and a sound code of ethics is important in these, why not in medicine? We ask why?

We trust, ere long, to see a just and honorable appreciation of the efforts of the good and wise men of the profession in behalf of medical morals, as the foundation of science and human happiness. Upon these very principles everything else good, is based; andpoliticians and others, claim the benefits of adhering to them, and receive honor for refusing to "affiliate" with those who scorn and defy them. Then why not grant what it so freely allowed in paralell cases?

"Another thing—there are a number of intelligent non-professional gentlemen who are advocates of Homœopathy—not for the sake of that absurdity in theory, but because, while subscribing cordially and heartily to the scientific principles of the regular profession, which our enemies have styled Allopathy—they believe that homœopathic preparations are pharmaceutical wonders. They say, that homœopathic "mother-tinctures" and their other concentrated remedies are superior—infinitely so—to those prepared by our pharmaceutists. These gentlemen are honest, and indulge in a false belief, much to the prejudice of the profession and scientific pharmaceutists. All this has been "set afloat" and encouraged by homœopathists. Will not some of our able and honest pharmaceutists take the matter in hand, and see that justice is done both the professions of medicine and pharmacy?"

Will our journals of pharmacy make a note of this?—Eds. Com.

"We are glad to see that the Local Editors pledge themselves never to 'compromise the truth to make errôr respectable.'"

"I am rejoiced to see your noble stand for the rights of the profession. There is a sore trouble besetting the prosperity of the profession in the matter of professional fees and fee bills. Our science is progressing rapidly, and still the scientific practitioner is infinitely worse paid than any other profession or trade. What are the causes of this want of appreciation of medical men, and this inability to secure adequate remueration for the hard study and toil in the profession to which they have and are giving their endeavors? Will not some of your able editors take the matter in hand? As surely as the Universe exists, not many decades will pass until such a state of things will drive the scientific and talented men from our ranks—in fact, this is already taking place. What ambitious, intelligent young man, with such a prospect before him, would become so foolish as to turn his attention to medicine? Medicine is being murdered! By whom, and for what?"

We trust some of our editors will give their views upon the topics suggested above. In our opinion, the only escape from the difficulties besetting the profession is (1st) to organize local medicine societies in every county; (2d) to require all regular practitioners to become members; (3d) to enforce the ethics without "favor or affection" whenever violated; (4th) to make medical proficiency and moral qualifications the only passports by which membership can be secured in our State and National Associations.—*Eds. Companion.*

"I am delighted with your plan of having associate and corresponding editors. You have been fortunate in selecting the ablest writers of the different States. They can and doubtless will make the COMPANION all that the profession could desire."

"Adhere to the law, tell the whole truth, and you need not fear anything. Every true man everywhere will sustain you, so long as you are right—to sustain you in error would injure you in the end."

"A desire on the part of either Faculty or Trustees to ignore the law, to refuse to investigate the truth, ought rightfully, and will destroy the character and usefulness of any institution."

